# *MiR-23a* modulates *X-linked inhibitor of apoptosi*s-mediated autophagy in human luminal breast cancer cell lines

**DOI:** 10.18632/oncotarget.21080

**Published:** 2017-09-19

**Authors:** Ping Chen, Yin-Huan He, Xing Huang, Si-Qi Tao, Xiao-Nan Wang, Hong Yan, Ke-Shuo Ding, Peter E. Lobie, Wen-Yong Wu, Zheng-Sheng Wu

**Affiliations:** ^1^ Li Shui People's Hospital, Nanjing, Jiangsu, China; ^2^ Department of Pathology, Anhui Medical University, Hefei, Anhui, China; ^3^ Institute of Life Sciences, Key Laboratory of Developmental Genes and Human Disease, Southeast University, Nanjing, China; ^4^ Cancer Science Institute of Singapore and Department of Pharmacology, National University of Singapore, Singapore, Singapore; ^5^ Tsinghua Berkeley Shenzhen Institute, Tsinghua University Graduate School at Shenzhen, Shenzhen, Guangdong, China; ^6^ Department of General Surgery, The First Affiliated Hospital of Anhui Medical University, Hefei, Anhui, China

**Keywords:** miR-23a, autophagy, XIAP, breast cancer

## Abstract

Autophagy is a conserved multi-step lysosomal process that is induced by diverse stimuli including cellular nutrient deficiency. *X-linked inhibitor of apoptosis* (*XIAP*) promotes cell survival and recently has been demonstrated to suppress autophagy. Herein, we examined regulation of *XIAP*-mediated autophagy in breast cancer cells and determined the underlying molecular mechanism. To investigate this process, autophagy of breast cancer cells was induced by Earle's balanced salt solution (EBSS). We observed discordant expression of *XIAP* mRNA and protein in the autophagic process induced by EBSS, suggesting *XIAP* may be regulated at a post-transcriptional level. By scanning several miRNAs potentially targeting *XIAP*, we observed that forced expression of *miR-23a* significantly decreased the expression of *XIAP* and promoted autophagy, wherever down-regulation of *miR-23a* increased *XIAP*expression and suppressed autophagy in breast cancer cells. *XIAP* was confirmed as a direct target of *miR-23a* by reporter assay utilizing the 3′UTR of *XIAP*. *In vitro*, forced expression of *miR-23a* promoted autophagy, colony formation, migration and invasion of breast cancer cell by down-regulation of *XIAP* expression. However, *miR-23a* inhibited apoptosis of breast cancer cells independent of *XIAP*. Xenograft models confirmed the effect of *miR-23a* on expression of *XIAP* and *LC3* and that *miR-23a* promoted breast cancer cell invasiveness. Therefore, our study demonstrates that *miR-23a* modulates *XIAP*-mediated autophagy and promotes survival and migration in breast cancer cells and hence provides important new insights into the understanding of the development and progression of breast cancer.

## INTRODUCTION

Autophagy is a cell self-degradation process for long-lived proteins and damaged organelles. Autophagy is present in cells at a low basal level and can be promoted by diverse stressful conditions, such as adaptation to starvation, oxidative or genotoxic stress, and elimination of pathogens [1, 2]. The role of autophagy in cancer development and progression remains ambiguous. Different studies have demonstrated that autophagic process may function in both a tumor suppressor and oncogenic role [3, 4]. *In vivo* depletion of the expression of autophagy-related genes, such as *BECN1* and *ATG5*, has been reported to lead to tumor cell survival [5–8]. In contrast, autophagy may enhance tumor cell survival, especially in oncogenic *RAS*-driven cancer [9–11]. Hence, a better molecular understanding of the autophagic process will assist in delineating its roles in cancer progression.

*X-linked inhibitor of apoptosis* (*XIAP*), is a member of the “*inhibitors of apoptosis*” (*IAP*) family and characterized by the presence of at least one baculovirus *IAP* repeat (BIR) structural domain [12]. *XIAP* possesses the most potent anti-apoptotic capacity by binding and inhibiting of caspases (caspase-3, 7, and 9) *in vitro* [13, 14]. *XIAP* protein levels are regulated both transcriptionally and post-transcriptionally [15–18]. Our recent study demonstrated that *XIAP* was able to suppress autophagic activity of diverse tumor cell types by the Mdm2-p53 pathway independent of its anti-apoptosis function [19, 20]. However, the mechanism involved in the regulation of *XIAP* in autophagy is unknown.

Recent studies have identified microRNAs (miRNAs) as novel regulators of autophagy [21–23]. miRNAs are short (19∼25 nucleotides) non-coding RNAs, which may act as negative regulators of gene expression by binding to a target mRNA, resulting in posttranscriptional or translational repression [24, 25]. To date, only a few of miRNAs have been reported to modulate autophagic activity directly. *MiR-30d* was observed to impair the autophagic process by targeting multiple genes in the autophagic pathway [26]. *miR-376a* regulates starvation-induced autophagy by controlling of *ATG4C* and *BECN1* transcript and protein levels [27]. *MiR-199a-5p* plays differential roles in radiation-induced autophagy in breast cancer cells by regulating the expression level of *DRAM1* and *BECN1* [28]. Herein, to investigate the regulatory mechanism of *XIAP* in cell autophagy, we scanned several miRNAs and identified *miR-23a* as a target miRNA for *XIAP*-mediated autophagy and also play a role in cell viability, invasion and migration of breast cancer.

## RESULTS

### Autophagy are associated with *XIAP*

**Figure 1:**
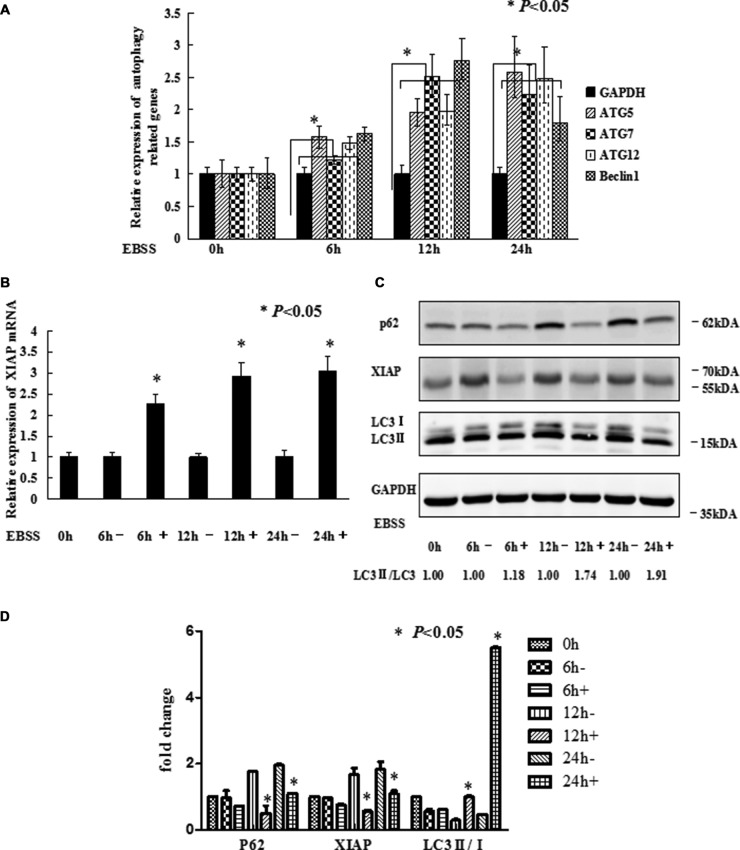
EBSS induces autophagy in breast cancer cells (**A** and **B**) MCF-7 cells at 80%–90% confluence were cultured with EBSS for 0, 6, 12, and 24 h, compared with normal medium. Cells were collected for qRT-PCR to quantify the expression level of the autophagy related genes and *XIAP*. The error bars indicate the standard error of the mean (S.E.M) for three independent experiments (*, *P* < 0.05). (**C**) Western blot analysis of *XIAP* and *LC3-II/I* expression after MCF-7 cells treated with EBSS. (**D**) The gray value of *P62*, *XIAP* and *LC3-II/I* was calculated.

**Figure 2:**
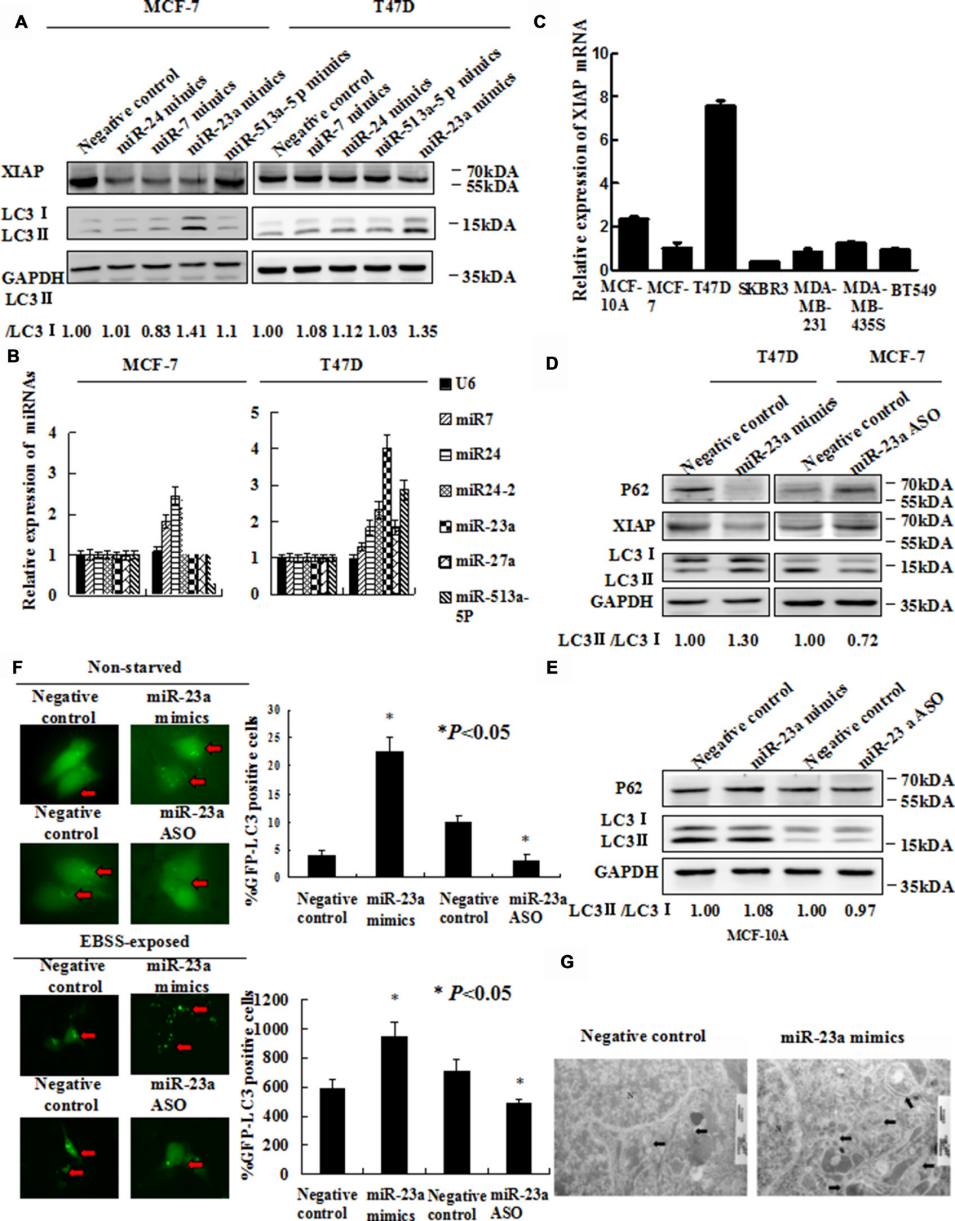
Forced expression of *miR-23a* induces autophagic activity (**A**) MCF-7 and T47D cells were transfected with *miR-24* mimics, *miR-7* mimics, *miR-23a* mimics and *miR-513a-5p* mimics. Forty-eight hours later, *LC3-II/I* proteins were detected by Western blot. (**B**) EBSS induced *miR-23a* expression strongly in MCF-7 and T47D cells. Cells were treated with EBSS and compared cells grown in with normal medium. Shown is the qRT-PCR analysis for *miR-24*, *miR-7*, *miR-513a-5p* and *miR-23a*. *U6* snRNA was used as an input control (*, *P* < 0.05). (**C**) *XIAP* mRNA expression in seven human mammary cell lines was analyzed by qRT-PCR. (**D**) Expression of *miR-23a* induces *LC3* conversion and *SQSTM1/P62* degradation. T47D cells were transiently transfected with *miR-23a* mimics and MCF-7 cells were transiently transfected with *miR-23a* ASO. Total cellular protein was isolated and subjected to Western blot analysis. *GAPDH* was used as input control. (**E**) Expression of *miR-23a* did not significantly induce *LC3* conversion and *SQSTM1/P62* degradation. MCF-10A cells were transiently transfected with *miR-23a* mimics and *miR-23a* ASO. Total cellular protein was isolated and subjected to Western blot analysis. *GAPDH* was used as input control. (**F**) GFP-*LC3* puncta formation was analyzed by fluorescence microscopy (200× magnification). Black arrows indicate clusters of GFP-*LC3* puncta in cells. Quantification of GFP-*LC3* puncta in E (mean ± S.D of independent experiments, *n* = 3,*P < 0.05). (**G**) Autophagy was evaluated in breast cancer cells by electron microscopy. Scale bars, 200 nm.

To trigger autophagy, MCF-7 cells were cultured with or without Earle's balanced salt solution (EBSS) in a time-dependent manner. As reported in our previous study, *XIAP* inhibited autophagy via *XIAP*-Mdm2-p53 signaling pathway [19]. QRT-PCR was performed to ensure the expression of *XIAP* and autophagy related genes including *ATG5*, *ATG7*, *ATG12* and *Beclin1*. As shown in Figure 1A, the expression levels of the autophagy related genes were all increased, which suggested that EBSS induced autophagy in these cells. Meanwhile, the expression level of *XIAP* mRNA was also increased (Figure 1B). To verify these results, we further analyzed the protein levels of *XIAP* and determined the conversion of *LC3-I* (cytosolic form) to *LC3-II* (membrane-bound lipidated form) by Western blot analysis. To our surprise, the protein level of *XIAP* was decreased, and *LC3-II/LC3-I* conversion ratio was increased, in the presence of EBSS (Figure 1C). The gray value we calculated as shown in Figure 1D. We also used 3-MA, an autophagy inhibitor, in the EBSS-exposed cells, and the expression level of *SQSTM1/P62* protein was increased and *LC3-II/LC3-I* conversion ratio was decreased. This result clarified the observed increase in *LC3-II* is suggestive of induction of autophagy (Supplementary Figure 1). Hence, there exists discordance between *XIAP* mRNA and protein levels in the process of autophagy, suggesting that *XIAP* may be regulated at a post-transcriptional level, potentially by miRNAs. To determine whether miRNAs potentially participated in regulating autophagy, we identified several miRNAs potentially targeting *XIAP* by bioinformatic analysis, including *miR-24*, *miR-7*, *miR-23a* and *miR-513a-5p*. Of these miRNAs, *miR-23a* not only significantly down-regulated the expression of *XIAP*, but also increased *LC3-II/LC3-I* conversion ratio in both MCF-7 and T47D cells (Figure 2A). We therefore selected *miR-23a* to further investigate the influence of miRNAs on *XIAP*-mediated autophagy.

### Overexpression of *miR-23a* enhanced autophagy

To explore the role of miRNAs in autophagy, we performed qRT-PCR analysis for the expression levels of *miR-24*, *miR-7*, *miR-513a-5p* and *miR-23a* in MCF-7 and T47D cells treated with EBSS. The level of *miR-23a* expression was the most increased of 4 miRNAs in cells cultured with EBSS, compared with cells cultured with normal medium (Figure 2B). By determination of *XIAP* expression in common mammary epithelial and breast cancer cell lines, we selected T47D and MCF-7, as a couple of model cell lines with relatively high and low expression of *XIAP* respectively (Figure 2C). We performed Western blot analysis to detect *LC3-II/LC3-I* conversion ratio in T47D and MCF-7 cells after transfection with *miR-23a* mimics and *miR-23a* ASO, respectively. *MiR-23a* mimics diminished the expression of *SQSTM1/P62* protein and increased *LC3-II*/LC3-I conversion ratio in T47D cells. In contrast, *miR-23a* ASO significantly increased the expression of *SQSTM1/P62* protein and decreased *LC3-II / LC3-I* conversion ratio in MCF-7 cells (Figure 2D). However, there is not a significant change about the expression of *SQSTM1/P62* and *XIAP* protein after transfection with *miR-23a* mimics or ASO in non-tumorigenic MCF-10A (Figure 2E). Next, *miR-23a* mimics and *miR-23a* ASO, respectively, were transfected into T47D and MCF-7 cells together with the GFP-*LC3* plasmid and examined by fluorescence microscopy. As shown in Figure 2F, there was a significant increase of GFP-*LC3* puncta in *miR-23a* mimics transfected cells and a decrease of GFP-*LC3* puncta in *miR-23a* ASO transfected cells both in non-starved and EBSS-exposed breast cancer cells. Consistent with the GFP-*LC3* puncta formation assay, we observed that accumulation of autophagosomes was increased in *miR-23a* mimics transfected cells by transmission electron microscopy (Figure 2G).

### *MiR-23a* directly targeted *XIAP 3′UTR*

**Figure 3:**
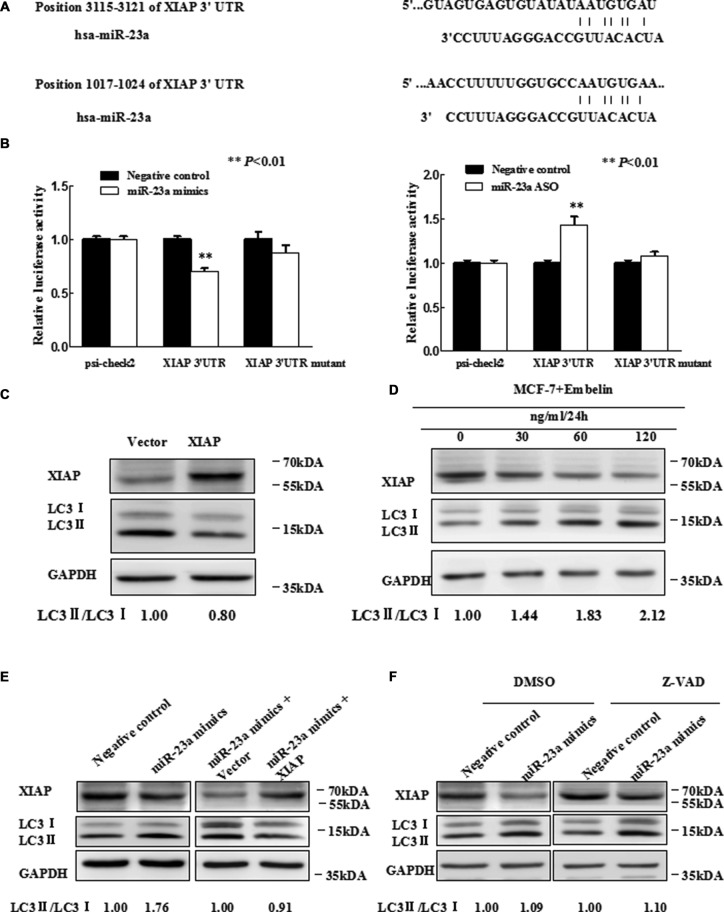
*MiR-23a* directly targets *XIAP 3′UTR* (**A**) Predicted binding sequences between *miR-23a* and seed matches in *XIAP-3′UTR*. (**B**) Luciferase reporter analysis of *XIAP 3′UTR* were performed after co-transfection with *miR-23a* in T47D cells and after co-transfection with *miR-23a* ASO in MCF-7 cells. The error bars indicate the standard error of the mean (S.E.M.) for three independent experiments (*P < 0.05). (**C**) Expression of *XIAP* inhibits autophagy. Western blot analysis is shown. Vector is used as an internal control. (**D**) MCF-7 cells were treated with Embelin as indicated and *XIAP* protein level and *LC3-II/I* expression were determined by Western blot analysis with anti-*XIAP* and anti-*LC3*, respectively. (**E**) Western blot. T47D cells were grown and transfected with *miR-23a* mimics, *miR-23a* mimics plus vector, *miR-23a* mimics plus expression of *XIAP*, or negative control. Then, total cellular proteins from these cells were subjected to Western blot analysis of *XIAP*, *LC3* expression. (**F**) Western blot. Forty-eight hours after transfected with *miR-23a* mimics and Negative control in T47D cells, which were treated with 20 uM Z-VAD-FMK or DMSO for 2 h. Cell lysates were analyzed by Western blotting.

Having established the function of *miR-23a* in autophagy, we next determined whether *miR-23a* directly targeted *XIAP*. Firstly, we identified two potential binding sites of *miR-23a* in the *XIAP-3′UTR* by Targetscan [29] (Figure 3A). We then performed qRT-PCR to examine the level of *XIAP* mRNA in cells transfected with *miR-23a* mimics and *miR-23a* ASO. As shown in Supplementary Figure 2, *miR-23a* produced a slight decrease in the *XIAP* mRNA level and *miR-23a* ASO exerted the opposite effect. Western blot analysis showed that the cellular levels of *XIAP* protein were significantly decreased in *miR-23a* mimics transfected cells and significantly increased in *miR-23a* ASO transfected cells (Figure 2D).

Luciferase reporter assay demonstrated that *miR-23a* directly interacted with *XIAP 3′UTR* and this interaction occurred at positions 3115-3121 (Figure 3B), but not positions 1017–1024 (data not shown). We next determined whether promotion of autophagy by *miR-23a* was mediated by *XIAP*. We first constructed a *XIAP* expression vector, pIRESneo3/*XIAP*, and the forced expression of *XIAP* was confirmed by qRT-PCR (Supplementary Figure 3) and Western blot analysis (Figure 3C). We also used Embelin, an inhibitor of *XIAP*, to verify the function of *XIAP* on autophagy, by Western blot analysis for *LC3-II/LC3-I* (Figure 3D). We observed that Embelin promoted autophagy in a dose-dependent manner (0–120 ug/ml). By transfection of *miR-23a* mimics, *miR-23a* mimics plus *XIAP* expression plasmid or controls, we observed that forced expression of *XIAP* significantly abrogated *miR-23a*-induced autophagy in T47D cells (Figure 3E).

For the essential role of *XIAP* in regulating cell apoptosis, we may ask that whether *miR-23a*-induced autophagy was associated with apoptosis. To this end, we transfected T47D cells with *miR-23a* mimics or a negative control followed by treatment with either caspase inhibitor Z-VAD-FMK or vehicle (DMSO). It was observed that the effect of *miR-23a* on *LC3-II/LC3-I* conversion ratio and the protein level of *XIAP* in T47D cells was not affected by Z-VAD-FMK treatment (Figure 3F), suggesting that *miR-23a* induced *XIAP*-mediated autophagy was independent of caspase-mediated apoptosis.

### Effects of *miR-23a* on breast cancer cell viability, migration, invasion and apoptosis

**Figure 4:**
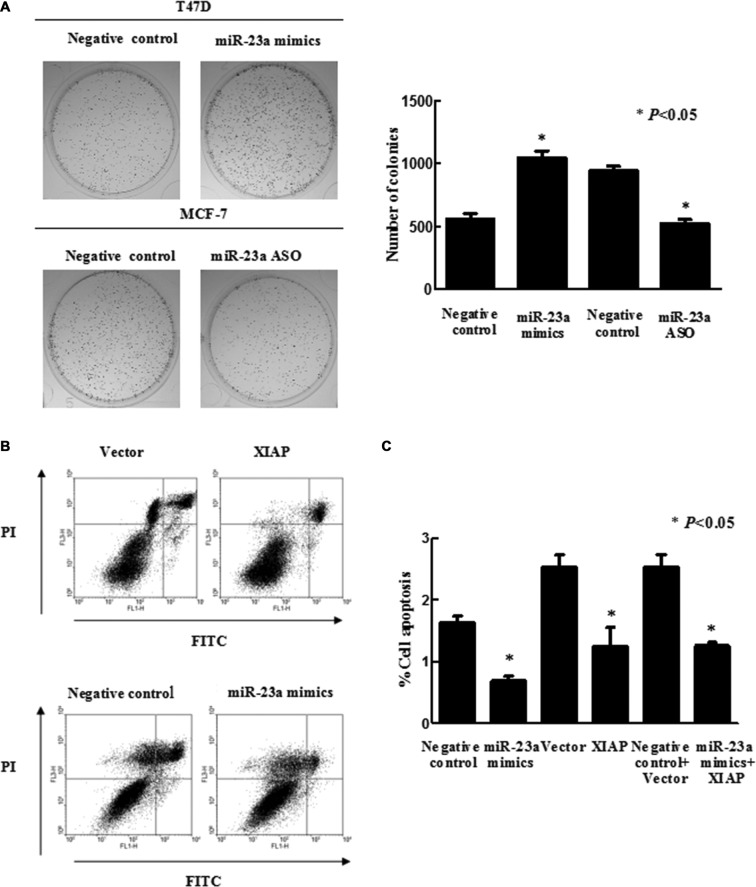
Effects of *miR-23a* on breast cancer cell viability and apoptosis (**A**) Colony formation assays. T47D and MCF-7 cells were grown and transiently transfected with *miR-23a* mimics and *miR-23a* ASO, respectively, and then seeded in 0.35% top agarose and 10% FBS in six-well plates in triplicate. The number of colonies was counted after 10 days incubation. (**B**) Flow cytometric analysis of apoptosis in T47D cells after genes transfection, as evidenced by PI/Annexin V double staining and FACS analysis. (**C**) The apoptosis rate was quantified by a flow cytometer. Data are presented as mean ± SEM (*n* = 3, *P < 0.05).

We next explored the effect of *miR-23a* on the behaviors of breast cancer cells *in vitro*. We observed that knockdown of *miR-23a* expression in MCF-7 cells by *miR-23a* ASO did not significantly alter cell viability (*P* > 0.05), and forced-expression of *miR-23a* in T47D cells by *miR-23a* mimics did not significantly reduce cell viability (*P* > 0.05) in MTT assay (data not shown). However, in a long-term cell survival assay, up-regulation of *miR-23a* expression by transfecting T47D cells with the specific mimics significantly increased cell colony formation, and downregulation of *miR-23a* expression by transfecting MCF-7 cells with the specific ASO significantly decreased cell colony formation (Figure 4A).

We next used transwell assays to assess the impact of *miR-23a* on cell migration and invasion. As shown in Supplementary Figure 4, T47D cells transfected with *miR-23a* mimics exhibited increased migration and invasion compared with controls. Moreover, co-transfection of cells with *miR-23a* mimics and the *XIAP* expression plasmid demonstrated that expression of *XIAP* significantly abrogated *miR-23a* mimic-promoted tumor cell migration and invasion (Supplementary Figure 4A). Furthermore, 48 h after transient transfection of *miR-23a* mimics, cells were treated with the autophagy inhibitor 3-MA for 24h. 3-MA significantly abrogated *miR-23a* mimic-promoted tumor cell migration and invasion (Supplementary Figure 4B). Consistently, MCF-7 cells transfected with *miR-23a* ASO exhibited decreased migration and invasion, compared with the control. Moreover, *XIAP* inhibitor Embelin and autophagy inducer EBSS dramatically abrogated *miR-23a* ASO-decreased tumor cell migration and invasion (Supplementary Figure 4C, 4D). These data implied that *miR-23a* promoted autophagy and cell migration and invasion using similar signaling pathways by regulation of *XIAP*.

Previous studies have demonstrated that *miR-23a* suppressed apoptosis in colorectal and gastric cancer cells [30, 31]. Consistently, we observed that *miR-23a* significantly inhibited apoptosis of breast cancer cells (Figure 4B). Next, we determined whether *XIAP* was involved in *miR-23a*-regulated apoptosis. To this end, we transfected *miR-23a* mimics, *miR-23a* mimics plus *XIAP* expression plasmid or controls into T47D cells and observed that forced-expression of *XIAP* did not abrogate the anti-apoptotic effect of *miR-23a* in these cells (Figure 4C).

### Effects of *miR-23a* on *XIAP* expression and tumor cell invasiveness in nude mouse xenografts

**Figure 5:**
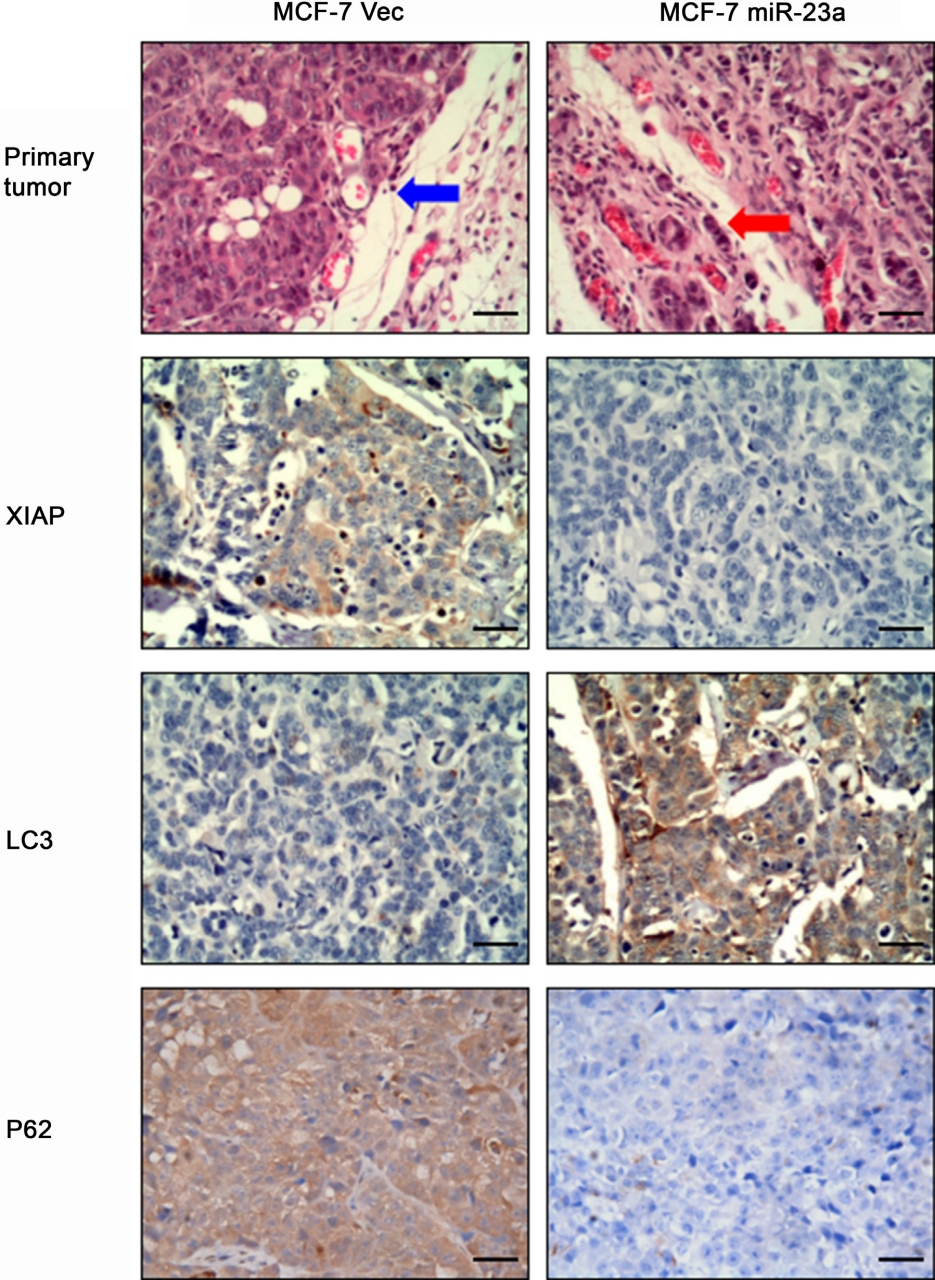
Effects of forced expression *miR-23a* on regulation of MCF-7 xenograft in nude mice MCF-7-Negative control and MCF-7- *miR-23a* cells were transplanted into the mammary fat pad of female BALB/c-nu, respectively. Hematoxylin and eosin staining of tumor xenograft sections. Invasive behavior was observed at the margin of the tumors generated by MCF-7-*miR-23a* mimics (red arrow) compared to that of MCF-7-Negative control cells (blue arrow). (Magnification: ×200). Representative imagines of *XIAP*, *LC3* and *P62* expression analyzed by immunohistochemistry (Magnification: ×200).

To determine the effect of *miR-23a* expression in breast cancer cells *in vivo*, we injected MCF-7-VEC and MCF-7-*miR-23a* cells orthotopically into the mammary fat pad of female BALB/c nude mice, respectively. Histology of xenografts showed that tumors derived from MCF-7-*miR-23a* cells were poorly encapsulated and highly invasive in comparison to tumors derived from control cells (Figure 5). More aggressive behavior was observed in the margin of tumor nodule of MCF-7-*miR-23a* cells (red arrow) compared to that of MCF-7-VEC cells (blue arrow). By use of immunohistochemistry, we also confirmed decreased protein expression of*XIAP*, *P62* and increased *LC3* expression in tumors generated by MCF-7-*miR-23a* cells compared to those generated by control cells (Figure 5).

## DISCUSSION

We have previously demonstrated that *XIAP* suppressed diverse cell autophagy independent of its regulation of apoptosis [19]. However, the upstream regulation of *XIAP*-mediated autophagy is unknown. We observed discordant expression of *XIAP* mRNA and protein after induction of autophagy which implied that *XIAP* may be post-transcriptionally regulated. We first identified *miR-23a* as a regulator of autophagy and demonstrated that *XIAP* is a target gene of *miR-23a*. Finally, we demonstrated that *miR-23a* enhanced breast cancer cell autophagic activity through modulation of *XIAP* expression and also promoted cell migration and invasion.

The function of autophagy in cancer development and/or progression is considered to be cell context-dependent [4, 32]. Autophagy ensures the delivery of metabolic substrates to cells so as to fulfill their energy demand during stress, thus supporting cell survival [33, 34]. However, hyperactivation of autophagy will result in cell death designated as ‘autophagic cell death’. Amino acid deprivation promotes autophagy in different organs and in cultured cells [35, 36]. Consistent with these reports, we have demonstrated that amino acid deficient increased autophagy activity in breast cancer cells.

It has been reported that the process of autophagy is modulated by miRNAs that regulate gene expression post-transcriptionally [21–23]. In the present study, we have identified *miR-23a* as a novel miRNA regulating basal autophagy. *MiR-23a* expression has been reported in a wide range of malignancies, including gastric, colorectal, and breast cancers [30, 31, 37]. Several lines of evidence suggest that *miR-23a* functions as an oncogene and is involved in tumor development. Herein, we demonstrated that forced expression of *miR-23a* inhibits apoptosis, promotes autophagy and enhances cell colony formation, migration and invasion. It was interesting to note that expression of endogenous *miR-23a* was increased in response to cellular stress caused by amino acid depletion. Transcriptional and/or post-transcriptional regulatory mechanisms may be responsible for increased *miR-23a* expression. In MCF-7 and T47D cell lines, we found that forced expression of *miR-23a* resulted in a significant increase *LC3-II* accumulation and *SQSTM1/P62* degradation. In addition, suppression of *miR-23a* by specific antagonism exerted the opposite effect. While there was no significant change concerning *P62* and *LC3-II/I* expression after transfection of *miR-23a* mimics or ASO in MCF-10A cell line. Our results support the idea that there were differences between *miR-23a* regulated cell lines. We further observed by transmission electron microscopy that accumulation of autophagosomes was increased in cells transfected with *miR-23a* and concordantly observed enhancement of GFP-*LC3* puncta formation by fluorescence microscopy.

One miRNA may modulate the expression of different target genes. By using bioinformatic analyses to search for potential target genes of *miR-23a*, we observed that *miR-23a* may directly target *XIAP* mRNA. Experimentally by use of reporter assays we observed that *XIAP* was indeed a target gene of *miR-23a* as expected. The mechanisms of *miR-23a*-promoted cell autophagy, survival, migration and invasion required further delineation. We demonstrated that increased *XIAP* expression significantly abrogated *miR-23a* mimics-promoted breast cancer cell autophagy, migration and invasion. Furthermore, 3-MA decreased *miR-23a* mimics-induced cell migration and invasion. However, the two processes of autophagy and cell migration/invasion may simply utilize similar signaling pathways and not be functionally or causally related 3-MA inhibits autophagy by acting as an inhibitor of *type III PI3K* [38, 39]. Enhanced *PI3K* activity has also been demonstrated to promote human fibrosarcoma cell migration and invasion [40]. Additionally, *XIAP* is the most potent caspase inhibitor of all *IAP* family members and has been observed to be increased in expression in a variety of human malignancies [41–45]. Hence we determined whether a potential functional interrelationship existed between *XIAP*-mediated autophagy and *XIAP* mediated cell survival. We demonstrated that *miR-23a* promoted *XIAP*-mediated autophagy was independent of the caspase-mediated apoptotic pathway. Interestingly, our data also revealed that *miR-23a* did indeed inhibit cell apoptosis but this function was not mediated by *XIAP*. The results are not surprising as one miRNA may modulate the expression of different target genes with a differential functional outcome dependent on the particular cellular context. For example, Xie et al. [16] showed that *miR-24* over-expression can overcome apoptosis-resistance in cancer cells via downregulation of *XIAP* expression. Liu et al. [30] demonstrated that *miR-23a* suppressed apoptosis of gastric cancer cells by targeting the *PPP2R5E* gene. Herein, suppression of breast cancer cell apoptosis by *miR-23a* is probably mediated by some other genes rather than *XIAP*.

Interestingly, the basal levels of *XIAP* in MCF-7 and T47D cells were discrepant, and the mRNA level of *XIAP* in T47D was nearly 8 times of that in MCF-7 (Figure 2C). As reported previously, many proteins involved in stimulation of cell growth, cell survival and cancer development were expressed more strongly in T47D than in MCF7 including for example, cyclin-D3 and prohibitin [46, 47]. Herein we consistently observed that the anti-apoptosis and anti-autophagic gene *XIAP* was also dramatically higher in T47D compared with MCF-7.

A compensatory mechanism that leads to increased expression of other *IAP* family members when *XIAP* expression is lost during apoptosis has been observed [48]. This work shed light on the independent relationship between autophagy and apoptosis mediated by *miR-23a*. It is reported that *miR-21*, *miR-155*, and *miR-221/222* may also mediate apoptotic and autophagic pathways of glioma cells, cervical cancer cells and breast cancer cells, respectively [49–52]. Similarly, we demonstrated that *miR-23a* promotes autophagy and inhibits apoptosis by different mechanisms in breast cancer cells.

## MATERIALS AND METHODS

### Cell culture and treatment

Human breast cancer cell lines MCF-7, T47D, SKBR3, BT549, MDA-MB-231, MDA-MB-435S and a human breast non-tumorigenic cell line MCF-10A were obtained from the American Type Culture Collection (ATCC, Manassas, VA, USA). All cells were maintained in a humidified incubator at 37°C and 5% CO_2_. Cells were treated with Earle's balanced salt solution (EBSS, Sigma) to activate starvation-induced autophagy [27]. Apoptosis inhibitor Z-VAD-FMK (Santa Cruz), autophagy inhibitor 3-MA (Sigma) and *XIAP* inhibitor Embelin (Santa Cruz) were treated when necessary.

### MiRNA transfection

Breast cancer T47D and MCF-7 cells (1.0 × 10^5^ /well) were seeded in 6-well plates overnight and then respectively transfected with *miR-23a* mimics (GenePharma, Shanghai) or its negative control and 2′-O methylated single-stranded *miR-23a* antisense oligonucleotide (ASO, GenePharma) or its negative control, and RNA duplex control using Lipofectamine 3000 (Invitrogen, Carlsbad, Calif, CA, USA) following the instructions of the manufacturer. The sequences of miRNA oligonucleotides were summarized in Supplementary Table 1.

### RNA extraction and quantitative real-time PCR (qRT-PCR)

Total cellular RNA and miRNA were isolated using Trizol reagent (Invitrogen) and the mirVana miRNA Isolation Kit (Ambion, Austin, TX), respectively, according to the manufacturer's introductions. QRT-PCR were performed to detect the expression of *XIAP*, *ATG5*, *ATG7*, *ATG12*, *Beclin1*, *miR-23a*, *GAPDH*, and *U6* as described previously [53–55]. The sequence of the primers used for qRT-PCR was summarized in Supplementary Table 2.

### Western blotting analysis

Total cellular protein and Western blotting analysis were performed according to pervious study [53, 54]. The antibodies used were as follows: anti-*XIAP* (E-2, Santa Cruz), anti-*LC3* (L7543, Sigma), anti-*SQSTM1/P62* (D-3, Santa Cruz), anti-*GAPDH* (A-3, Santa Cruz).

### Immunohistochemistry assay

For analysis of *XIAP* expression and *LC3* expression in tumors from nude mouse, a mouse anti-*XIAP* polyclonal antibody (H-202, Santa Cruz, 1:15) and a rabbit anti-*LC3* polyclonal antibody (L7543, Sigma, 1:100) were used according to our previous study [53–55].

### FACS analysis

Cell apoptosis was assayed using Annexin V- Apotosis Detection kit (BestBio, Shanghai, China) according to the manufacturer's introductions. All the experiments were performed using a FACScalibur cytometer (BD Biosciences, San Jose, CA). Each experiment was performed in triplicate and repeated at least once.

### GFP-*LC3* Localization assay

In order to generate expression of GFP-*LC3* in MCF-7 cells, we transiently expressed *miR-23a* and *miR-23a* ASO with the autophagy marker GFP-*LC3*, compared with negative control, 48h after co-transfection, GFP-*LC3* puncta were visualized under a fluorescence microscope (Olympus XSZ-D2) equipped with CCD cameras and images were captured and analyzed for presence of more than five puncta per cell.

### Electron microscopy

Cells were treated as indicated and fixed with 2.5% glutaraldehyde containing 0.1 mol/L sodium cacodylate. Samples were fixed using 1% osmium tetroxide, followed by dehydration with an increasing concentration gradient of ethanol and propylene oxide. Samples were then embedded, cut into 50nm sections, and stained with 3% uranyl acetate and lead citrate as previously reported [19]. Images were acquired using a JEM-1200 electron microscope (JEOL, Tokyo, Japan).

### Luciferase reporter assay

Cells were plated on a 24-well plate 24h before transfection at 50% confluence and then co-transfected with 0.2 ug of psiCHECK2-*XIAP 3′UTR* or psiCHECK2 control vector and 30 nM *miR-23a* mimics or its negative control by using Lipofectamine 3000. 48h after transfection, cells were harvested, and reporter assays were performed using a dual luciferase assay system (Promega). Each transfection was performed in triplicate. The primers for *XIAP 3′UTR* were 5′-GCGCGCACTCGA GTCTAACTCTATAGTAGGCATGTTATG-3′ (sense) and 5′-TATATGCGGCCGCCTACAATGAATGCCAGA TTATACAGC-3′ (antisense).

### MTT assay and colony formation assay

Cells were cultured in 96-well plates at 5000 cells per well, 24h after transfection. The 3-(4, 5-dimethylthiazol-2-yl)-2, 5-diphenyl-tetrazolium bromide (MTT) assay was used to determine cell viability 24 h, 48 h, 72 h, and 96 h after the cells were seed. Absorbance at 570 nm was measured using an automatic microplate reader. (Infinite M200; Tecan, Grodig, Austria). Next, the cells were cultured for 10 days, and colonies were counted. The experiment was performed in triplicate. Data are expressed as mean ± standard deviation (SD).

### Cell migration and invasion assay

To determine whether the effect of *miR-23a* on breast cancer cell migration and invasion was medicated by *XIAP in vitro*, we used a Transwell insert (8 μm, Corning, NY). T47D cells were transfected with negative control, *miR-23a* mimics and *miR-23a* mimics plus plasmid *XIAP* or an autophagy inhibitor, 3-MA. Meanwhile, negative control, *miR-23a* ASO and *miR-23a* ASO plus *XIAP* inhibitor Embelin or EBSS were transfected in MCF-7 cells. Transwell assays were performed as described previously [53–55]. Five macroscopic areas were selected randomly and counted the cell numbers. All experiments were experiment in triplicate.

### Nude mouse breast cancer cell xenograft assay

All animal work was performed according to Institutional Animal Care and Use Committee guidelines (available at www.iacuc.org) with local institutional approval. Briefly, the 5-week-old female BALB/c nude mice (Hunan SJA Laboratory Animal Co., Ltd.) were used for studies. 5 × 10^6^ MCF-7-VEC and MCF-7-*miR-23a* cells were suspended in 120 μl Matrigel /PBS at a ratio of 1:1 (v/v) and then injected into the mammary fat pad of female BALB/c-nu. One estrogen pellet was implanted into each mouse before injection. When animals were sacrificed, primary tumors were harvested for further analysis.

### Bioinformatic analysis and statistical analysis

The miRNA database TargetScan (release 5.1, http://www.targetscan.org/) was used to predict the targeting miRNAs of *XIAP*. Statistical evaluation was shown as means ± standard deviation (SD). Date was analyzed by SPSS 16.0 software. Differences between groups were compared using Student *t* test for continuous variables. *P* values were considered significance if *P* < 0.05.

## SUPPLEMENTARY FIGURES AND TABLES


