# Supplementary material for: MiR-23a modulates X-linked inhibitor of apoptosis-mediated autophagy in human luminal breast cancer cell lines
Source: Oncotarget. 2017 Sep 19;8(46):80709–21. doi: 10.18632/oncotarget.21080 (PMC5655233; doi:10.18632/oncotarget.21080)
Supplement: Supplementary file 1 [file oncotarget-08-80709-s001.pdf]

MiR-23a modulates *X-linked inhibitor of apoptosis*-mediated autophagy in human luminal breast cancer cell lines

SUPPLEMENTARY MATERIALS

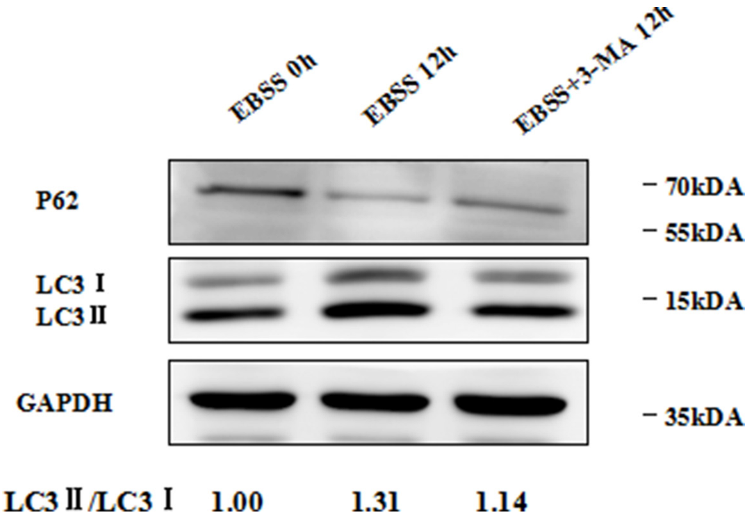

Supplementary Figure 1: *LC3-II* is suggestive of induction of autophagy. Western blot analysis of *SQSTM1/P62* and *LC3-II/I* expression after MCF-7 cells treated with EBSS and 3-MA.

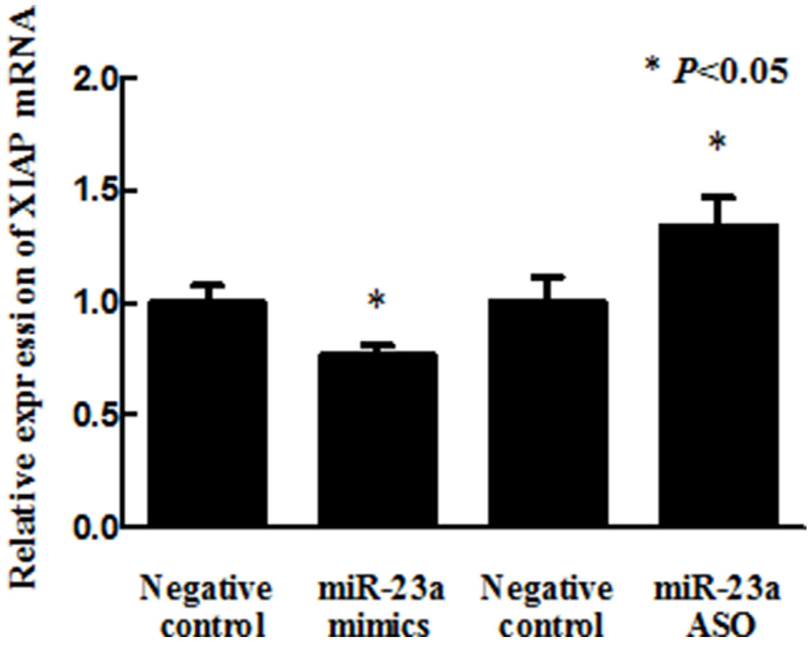

Supplementary Figure 2: QRT-PCR analysis of *XIAP* expression. T47D and MCF-7cells were culture and transiently transfected with *miR-23a* mimics and *miR-23a* ASO, respectively, and then perform RNA extraction and qRT-PCR, *$P > 0.05$.

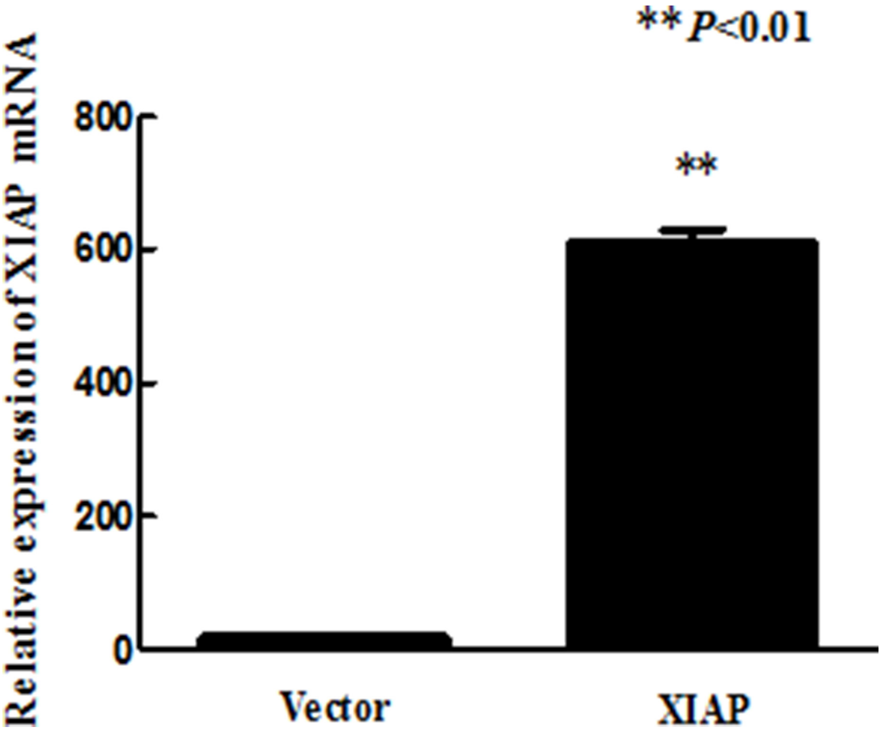

Supplementary Figure 3: QRT-PCR analysis of *XIAP* expression. T47D cells were transfected with *XIAP* expression plasmid, Vector was used as an internal control. The results are presented as the means ± SD of values obtained in 3 independent experiments, $^{**}P < 0.01$.

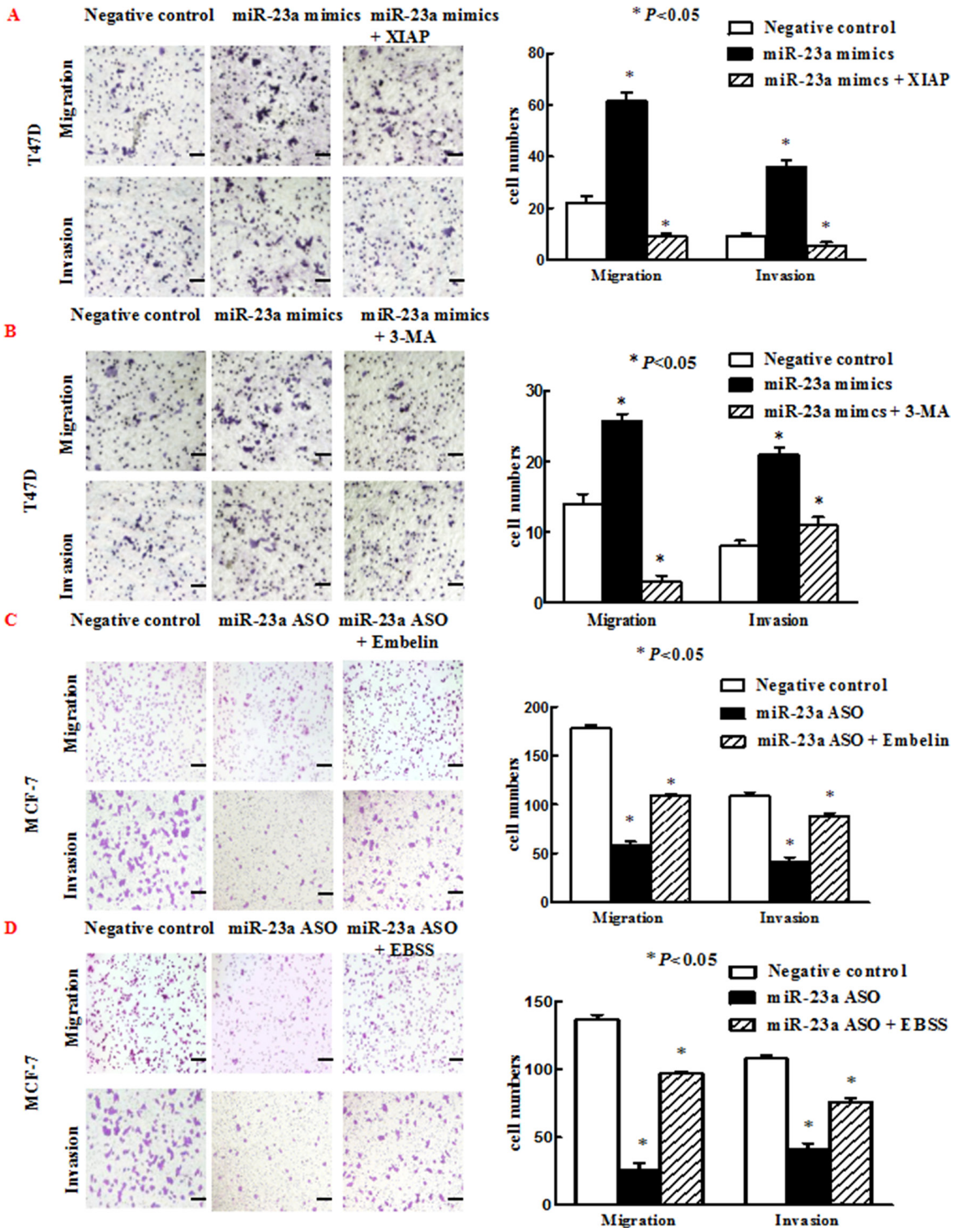

Supplementary Figure 4: Transwell migration and invasion are shown. (**A**) T47D cells were transfected with *miR-23a*, *miR-23a* mimics plus expression of *XIAP* or negative control for 48 hours and subjected to migration and invasion assays. (**B**) T47D cells were transfected with *miR-23a* or negative control and 48 hours later treated with 5 mM 3-MA for 24 h and subjected to migration and invasion assays. (**C**) MCF-7 cells were transfected with *miR-23a* ASO or negative control and treated with Embelin and subjected to migration and invasion assays. (**D**) MCF-7 cells were transfected with *miR-23a* ASO or negative control and treated with EBSS and subjected to migration and invasion assays. Representative photographs (left) and quantification (right) are shown. Original magnification ×200.

Supplementary Table 1: Sequences of miRNA oligonucleotides

miRNA	Sense Strand (5′-3′)	Antisense Strand (5′-3′)
miR-23a mimics	5′-AUCACAUUGCCAGGGAUUUCC-3′	5′-AAAUCCCUGGCAAUGUGAUUU-3′
Negative control	5′- UUCUCCGAACGUGUCACGUTT-3′v	5′-ACGUGACACGUUCGUAGAATT-3′
miR-23a ASO	5′ -GGAAAUCCCUGGCAAUGUGA- 3′	
ASO NC	5′-CAGUACUUUUGUGUAGUACAA-3′	

Supplementary Table 2: Sequences of the oligonucleotide primers used for qRT- PCR are as follows

Name	Sequence 5′-3′
GAPDH sense	5′- TGCACCACCAACTGCTTAGC-3′
GAPDH antisense	5′-GGCATGGACTGTGGTCATGAG-3′
ATG5 sense	5′-AGAATAGCCAGTACAGCA-3′
ATG5 antisense	5′-ATGAACCGACGAATAAAC-3′
ATG7 sense	5′-GTTGTTTGCTTCCGTGAC-3′
ATG7 antisense	5′-TGCCTCCTTTCTGGTTCT-3′
ATG12 sense	5′-GCGAACACGAACCATCCA-3′
ATG12 antisense	5′-CCACGCCTGAGACTTGCA-3′
Beclin1 sense	5′-CAA GAT CCT GGA CCG TGT CA-3′
Beclin1 antisense	5′-TGG CAC TTT CTG TGG ACA TCA-3′
XIAP sense	5′- CAAGAAATCCATCCATGGCAG-3′
XIAP antisense	5′- CAGTTAGCCCTCCTCCACAGTG-3′
U6 RT primer	5′-GTCGTATCCAGTGCAGGGTCCGAGGTGCACTGGATACGACAAAATATGG-3′
U6 forward primer	5′-GCTTCGGCAGCACATATACTAAAAT-3′
U6 reverse primer	5′- CGCTTCA CGAATTTGCGTGTCAT-3′
miR-23a RT primer	5′-GTC GTA TCC AGT GCA GGGTCC GAGGTA TTC GCA CTGGAT ACG AC GGAAAT -3′
miR-23a forward primer	5′-GCCGCATCACATTGCCAGGGA-3′
miR-23a reverse primer	5′-GTGCA GGGT CCG AGG T -3′
